# Intra-Abdominal Actinomycosis Mimicking Malignant Abdominal Disease

**DOI:** 10.1155/2017/1972023

**Published:** 2017-02-19

**Authors:** Ali Ridha, Njideka Oguejiofor, Sarah Al-Abayechi, Emmanuel Njoku

**Affiliations:** ^1^University of Arkansas for Medical Science, 4301 West Markham Street, Little Rock, AR 72205, USA; ^2^Chicago Medical School, Rosalind Franklin University of Medicine and Science, 3333 Green Bay Rd., North Chicago, IL 60064, USA

## Abstract

Abdominal actinomycosis is a rare infectious disease, caused by gram positive anaerobic bacteria, that may appear as an abdominal mass and/or abscess (Wagenlehner et al. 2003). This paper presents an unusual case of a hemodynamically stable 80-year-old man who presented to the emergency department with 4 weeks of worsening abdominal pain and swelling. He also complains of a 20-bound weight loss in 2 months. A large tender palpable mass in the right upper quadrant was noted on physical exam. Laboratory studies showed a normal white blood cell count, slightly decreased hemoglobin and hematocrit, and mildly elevated total bilirubin and alkaline phosphatase. A CT with contrast was done and showed a liver mass. Radiology and general surgery suspected malignancy and recommended CT guided biopsy. The sample revealed abundant neutrophils and gram positive rods. Cytology was negative for malignancy and cultures eventually grew actinomyces. High dose IV penicillin therapy was given for 4 weeks and with appropriate response transitioned to oral antibiotic for 9 months with complete resolution of symptoms.

## 1. Introduction

Actinomycosis is a rare chronic infectious disease caused by* Actinomyces israeli*, an aerobic or microaerophilic gram positive bacteria, present in the oral cavity, throughout the gastrointestinal tract, female genital tract, and the bronchus [1]. Actinomyces has low virulence; consequently disease occurs when the mucosal barrier has been compromised or in patients who are immune compromised [2]. Diagnosis preoperatively is rarely made due to variable clinical presentations. The majority of cases are diagnosed after the specimen in question has been resected and examined histologically [3].

Abdominal actinomycosis develops after a localized inflammatory process, prolonged IUD use, or recent abdominal surgery [4]. The appendix, cecum, and colon diverticulum are most affected. It is characterized by infiltrative and granulomatous inflammation similar in presentation to irritable bowel disease, tuberculosis, and malignancy macroscopically [4].

The direct extension of actinomyces, across the tissue, leads to formation of multiple abscesses, abundant granulation tissue, and sinuses [5]. The involvement of surrounding structures not only contributes to the insidious clinical course and delay of diagnosis, but also may mimic a tumor. This paper presents a case of intra-abdominal actinomycosis mimicking malignant abdominal disease.

## 2. Case Report

80-year-old man with a known past medical history of atrial fibrillation, hyperlipidemia, benign prostatic hyperplasia, osteoarthritis, and laparoscopic cholecystectomy in 2009 presented to the emergency department with a four-week history of abdominal pain and swelling localized to the right upper quadrant (RUQ). Patient reported that the abdominal pain was dull, aching, nonradiating, 7/10 in severity, aggravated by movement, alleviated by rest, and associated with loss of appetite and 20 pounds' weight loss in 2 months. He denied any associated fever.

Physical examination revealed an elderly male who appeared to be in mild painful distress. Vital signs were as follows: temperature 97.8°F, pulse 89/bpm, respirations 16/min, blood pressure 100/69 mm/Hg, and oxygen saturation 98% on room air. Abdominal exam revealed well healed old laparoscopic scars. There was a large palpable mass in the RUQ which was tender to palpation. There was no palpable hepatosplenomegaly, guarding or rebound with normal active bowel sounds. The rest of his exam was unremarkable.

Laboratory studies demonstrated a white blood cell count of 8.3 10^3^/mcL, hemoglobin level 12.3 gm/dL, platelet count 315 10^3^/mcL, and mean corpuscular volume 99.2 FL (82–99). Liver function tests were normal; alpha fetoprotein was normal at 1 ng/mL, and carcinoembryonic antigen was also unremarkable at 1.2 ng/mL.

**Figure 1:**
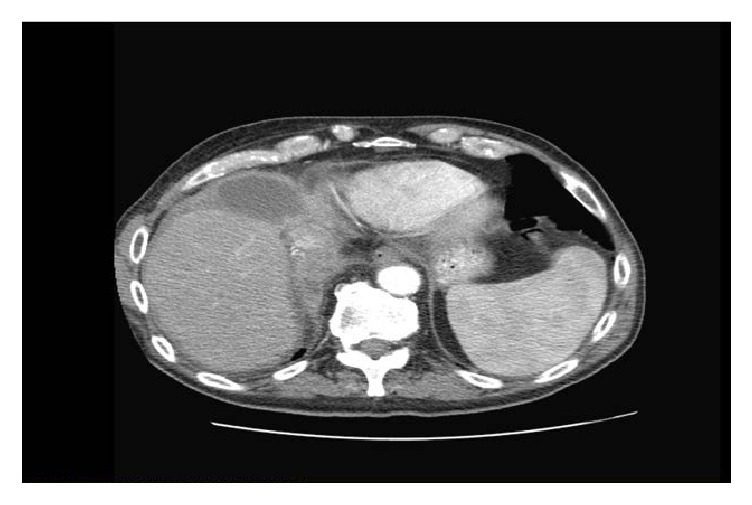
CT scan with contrast showing 5.6 cm × 2.2 cm liver mass and 3.7 cm × 2.2 cm fluid collection posterior to the anterior abdominal wall musculature.

Computerized tomography with contrast showed septated subpulmonic mass measuring 5.6 cm × 2.2 cm with internal high density anterior to the liver and adjacent to the diaphragm and 3.7 cm × 2.2 cm fluid collection posterior to the anterior abdominal wall musculature in the RUQ extending to the right rectus muscle (Figure 1). The radiologist suspected a malignancy with large hematoma knowing that he is chronically on Coumadin for atrial fibrillation. General surgery was called for evaluation and the surgeons recommended a CT guided biopsy of the mass and aspiration of the fluid collection. His Coumadin was held and a CT guided biopsy and aspiration were done with about 100 cc of thick viscus fluid drained. Fluid revealed abundant neutrophils and gram positive rods. Cultures eventually grew actinomyces and cytology was negative for any malignancy.

He was started on high dose intravenous penicillin G 3000000 units' every 4 hours for about 4 weeks with appropriate response. He was then transitioned to oral penicillin for about 9 months with complete resolution of symptoms.

## 3. Discussion

Actinomycosis is an infection caused by* Actinomyces* species.* Actinomyces* are gram positive, filamentous, nonsporing, and microaerophilic or obligate anaerobic bacteria. These bacteria normally colonize the flora of the oral cavity, genital tract, and upper gastrointestinal tract. They have a granulomatous inflammatory response causing pus production and abscess formation which is then followed by necrosis and extensive, reactive fibrosis [1]. The incidence of* Actinomyces* is 1 : 300000 [6]. Overall incidence of actinomycosis has been decreasing; however, abdominal and genital actinomycosis has been noted to be increasing in frequency due to increase of usage of intrauterine device [6].

Abdominal actinomycosis accounts for 20% of actinomycosis infection [7]. This particular type can occur due to a destruction of mucosal barriers, including perforated bowel, endoscopic procedures (like in our case), trauma, appendectomy, or due to an unknown cause [8]. One important challenge with actinomycosis infection is delayed diagnosis. It may present as a malignant disease, with symptoms of abdominal pain, asthenia, and weight loss.

Histopathologically, the organism produces the characteristic granulomatous inflammatory response. Confirmation is done by FNA or core biopsy by surgical exploration or radiological guided biopsy. Radiological techniques, including CT scan or magnetic resonance imaging (MRI), may show findings suggestive of the diagnosis of actinomycotic mass, in the right clinical setting. CT shows low-attenuation, focal areas of a solid mass and less frequently a thickened-wall cystic mass [9]. Actinomycosis treatment is centered on high dose antibiotics, including the standard treatment of 2–6 weeks IV penicillin G, followed by 6–12 months of oral penicillin [10].

## 4. Conclusion

Abdominal actinomycosis is an uncommon infectious disease that can mimic multiple disease processes. It may present as a malignant disease, with symptoms of abdominal pain, asthenia, and weight loss. High index of suspicion is needed to avoid delay in diagnosis. Confirmation is done by FNA or core biopsy by surgical exploration or radiological guided biopsy. In many patients prolonged treatment of high dose penicillin is required to be cured.
